# Seasonal sensitivity and psychiatric morbidity: study about seasonal affective disorder

**DOI:** 10.1186/s12888-021-03313-z

**Published:** 2021-06-29

**Authors:** Aníbal Fonte, Bruno Coutinho

**Affiliations:** 1 Department of Psychiatry and Mental Health / Local Health Unit of the Alto Minho (ULSAM, EPE), Hospital de Santa Luzia, Estrada de Santa Luzia, 4901-858 Viana do Castelo, Portugal; 2 Department of Clinical Neuroscience and Mental Health, Faculty of Medicine, University Porto, Porto, Portugal; 3 CINTESIS-UP, Hospital de Santa Luzia, Estrada de Santa Luzia, 4901-858 Viana do Castelo, Portugal; 4 Local Health Unit of the Northeast (ULSNE, EPE), Bragança, Portugal

**Keywords:** Seasonal affective disorder, Seasonal pattern assessment questionnaire, Seasonality, Psychiatric morbidity

## Abstract

**Background:**

Seasonal Affective Disorder is a recurrent depressive disorder which usually begins in the fall/winter and enters into remission in the spring/summer, although in some cases may occur in the summer with remission in the autumn-winter. In this study the authors evaluated the association between seasonal changes in mood and behavior with psychiatric disturbance.

**Method:**

Descriptive, cross-sectional study. Participants, students attending higher education and vocational courses (*N* = 324), were evaluated with the Seasonal Pattern Assessment Questionnaire (SPAQ) and the Screening Scale for Mental Health (ER80).

**Results:**

Among the respondents, 12.7% showed seasonal affective disorder (SAD), 29.0% showed subsyndromal seasonal affective disorder (s-SAD) and 58.3% did not show significant seasonal affective symptomatology. As for psychiatric morbidity, 36.6% of subjects with SAD and 13.8% of those with s-SAD were considered “psychiatric cases” whereas for subjects without SAD this value was only 3.2%.

**Conclusions:**

There is a statistically significant association between psychiatric morbidity and seasonal affective disorder. This association corroborates the importance of the Seasonal Pattern Assessment Questionnaire in screening for seasonal fluctuations in mood and behavior related disorders, and the clinical need for recognition of these conditions, particularly associated suffering and disabilities.

**Supplementary Information:**

The online version contains supplementary material available at 10.1186/s12888-021-03313-z.

Seasonal affective disorder (SAD) is a complex entity, defined by Rosenthal et al., in 1984, as a syndrome characterized by recurrent depressive episodes in autumn or winter, with remission in the spring or summer, occurring for at least two consecutive years, not explained by any variation in psychosocial factors [1].

Although seasonal variations in mood and behavior have been recognized since antiquity, their identification as a psychiatric disorder remains controversial, and the most recent review of the Diagnostic and Statistical Manual of Mental Disorders (DSM-5) only recognizes seasonal manifestations of mood as a “*seasonal pattern*” specifier for subgroups of patients with bipolar and recurrent major depressive disorders [2], while the 10th revision of the International Statistical Classification of Diseases and Related Health Problems (ICD-10) [3, 4], does not recognize it directly, classifying it as a recurrent depressive or bipolar disorder, arguing that more studies are needed before it can be internationally accepted.

As for the manifestations, SAD shares with depression the nuclear symptoms of depression (depressed mood, lack of energy, loss of ability to feel pleasure or interest). However, although not specific, there is often a reversal in the pattern of the vegetative symptoms of depression that bring it closer to atypical depression, with increased sleep duration, increased appetite and weight gain, and altered eating habits with craving for carbohydrate based foods [1, 5–7]. In the most characteristic form, seasonal cycles emerge in autumn-winter but in some cases may occur in summer with remission in autumn-winter [6, 8, 9]. However summer SAD is not only less frequent but the symptoms are closer to non-seasonal depressive symptoms, with insomnia, poor appetite, weight loss, agitation, anxiety [9, 10]. In contrast to SAD, other forms of recurrent depression, like bipolar or unipolar depression, occur independently of the time of year [2].

Another point to consider is the variability of symptoms intensity. On one hand, there are seasonal variations without clinically significant symptomatology within the normal patterns of mood variability. On the other hand there are seasonal affective disorders with extremely disabling manifestations, such as those experienced by patients with disorders similar to severe recurrent major depressive episodes or bipolar affective disorder type II, with phases of hypomania in the spring or summer, while phases of mania are rare [1, 6, 11, 12], but clinical and epidemiological studies on the situations are more scarce.

As a “complex” disorder to which genetic, biochemical, environmental, and cultural factors contribute [10, 13, 14], studies on the prevalence of SAD show very different results to which several factors contribute, such as criteria and the evaluation instruments used, the region (latitude, weather conditions) where the studies are carried out or characteristics of the studied sample (gender, age, ethnic groups, acclimatization, attitudes towards mental manifestations, vulnerability to stress) [6, 7, 15–17]. Winkler and Kasper, in an article in which they gathered 22 studies, point to a prevalence of SAD in temperate countries between 2 and 5% [18]. But other studies estimate that the prevalence of SAD in populations located in northern latitudes can approach 10% [19] (12% in Copenhagen) [20]. Reflection of this diversity of results, Magnusson in a review and systematize epidemiological studies of SAD provided prevalence estimates ranging from 1.4 to 9.7% in Northern America, 1.3–3.0% in Europe and 0–0.9% in Asia [17]. In women, the prevalence tends to be higher than men, with a ratio varying between 3.5: 1 and 9:1 and, in most cases, the first episode of seasonal affective disorder occurs around the age of 30, with a decrease of discrepancy between the sexes with increasing age [18, 19].

Two issues are central to the conceptualization and operationalization of SAD. First, SAD is not a simple nosological entity. The seasonal pattern of recurrences, in terms of both symptomatology and intensity of seasonal manifestations, lies in what N. Rosenthal describes as a *“spectrum disturbance”* [21], depending on where the person is in that spectrum to qualify as having SAD or a milder form called the winter blues or subsyndromal seasonal affective disorder (s-SAD) [21, 22].

Second, despite being recognized as a syndrome, SAD does not exist as a specific diagnostic category and the criteria used to identify it have different amplitudes, and even restrictive criteria such as those present in the DSM-5 and ICD-10 lead to divergent results [23].

One of the most used instruments in epidemiological surveys is the Seasonal Pattern Assessment Questionnaire (SPAQ) developed by Rosenthal et al. [24] Translated and adapted for numerous languages ​​and communities, it was designed to recognize the sensitivity of individuals to seasonal variations on a *continuum,* regardless of the presence of other psychopathological conditions, both in clinical and nonclinical populations, and their reliability, internal consistency, temporal stability and validity, demonstrated in several studies [6, 25–28]. However, it has also been the subject of criticism, in particular because it leads to results that may overestimate the prevalence of seasonal depression [29–31] and its usefulness is limited to estimating the seasonal sensitivity of mood and behavior in individual health.

In this context, the objective of this work is to investigate the association between the seasonal sensitivities identified as SAD and s-SAD, regardless of the seasonal pattern in which it occurs, and the self-perceived state of mental health with the hypothesis that cases traced by the SPAQ correspond significantly to clinically important psychiatric morbidity situations.

## Methodology

### Study participants

The sample consisted of university students from various courses (medicine, rehabilitation, nursing, engineering) and non-university students enrolled in vocational training programs, from the north of Portugal (Porto, Viana do Castelo), in order to gather a heterogeneous sample of both genders, with different levels of schooling and age distribution. The study was carried out throughout the school year, predominantly from February to May and October to November.

### Instruments

In addition to a questionnaire for recording demographic data, we used:

Seasonal Pattern Assessment Questionnaire (SPAQ). An instrument developed by Rosenthal et al., translated and measured in Portuguese [28]. This is a questionnaire for retrospective evaluation of mood and behavior changes over time. It is organized into six sections, although only two sections are considered in this study, which form the core of the questionnaire. The first of these sections is made up of six items (sleep duration, social activity, mood, weight, appetite, energy level) punctuated on a five-point scale, from “not changing” (0 points) to “changing a lot” (4 points), according to the susceptibility to changes with the season. The sum of the six items, in a total of 0 to 24 points, constitutes the Global Seasonality Score (GSS), where higher scores correspond to greater sensitivity to seasonal changes. In the second part, respondents indicate whether seasonal changes are a problem and, if so, its intensity, from “light” (1 points) to “disabling” (5 points).

The combination of scores attributed to the Global Seasonality Score (GSS) with the evaluation of seasonal changes as a problem allows us to define three types: “without seasonal affective disorder” (without SAD), “subsyndromal seasonal affective disorder” (s-SAD), “Seasonal affective disorder” (SAD). In this typology, according to the so-called Kasper criteria [22, 32], “without seasonal affective disorder” (without SAD) is considered when the Global Seasonality Score is < 9 or equal to 9 or 10 and the seasonal changes are not a problem or are mild (=1). The “subsyndromal seasonal affective disorder” (s-SAD) (defined as a cluster of seasonal symptoms with insufficient intensity to allow the diagnosis of seasonal affective disorder) is recognized when GSS = 9 or 10 and the seasonal changes scored as equal problem or higher than moderate (≥2), or GSS ≥ 11 and seasonal changes are not a problem or have a low to moderate level (0 or 1). In the third group, it is classified as “seasonal affective disorder” when GSS ≥ 11 and seasonal changes are a problem rated as equal to or greater than moderate (≥2). This combination of scores is often used in epidemiological studies even though the criteria are not always the same, particularly to distinguish between s-SAD and SAD and in how to value the response to the perception of seasonal changes as a problem [15].

The factorial structure of the Portuguese version of SPAQ was consistent with that of other studies, with the definition of two main factors, but the fact that the second factor has a eigenvalue < 1 (15,2% of variance) [28] led to the conclusion of the unidimensionality of the Global Seasonality Score subscale (GSS), in which each item evaluates different aspects of the same construct [25, 28, 33]. Cronbach’s alpha for the Global Seasonality Score subscale was 0.804 [28].

Screening Scale for Mental Health (ER80) (Pio de Abreu and Vaz Pato, 1981). Psychiatric morbidity scale with 16 self-assessment questions, addressed to physical and mental health, very simple to answer (example: have you had headaches? does it cost you to decide on the little things of everyday life?), scored 0 or 1, adapted to the Portuguese population [34]. Unifactorial scale, is intended to measure a widespread disturbance of the body’s homeostasis associated with disorders that characterize psychiatric illness. The total score ranges from 0 to 14 and a score higher than the threshold defined by the authors of the instrument (female gender ≥6, male gender ≥7) allows the identification of “psychiatric cases”. The scale includes five falsehood issues and a score of 4 or higher is suggestive of insincerity. The questionnaire has been widely used both in clinical (hospital and primary care) and non-clinical (students, emigrants, industrial workers) populations, with good discriminative indexes and internal consistency [35, 36]. The sensitivity and specificity calculated at validation were 86 and 87% respectively for females and 86 and 76% respectively for males [35]. In this study, Cronbach’s alpha of the ER80 was 0.785.

### Statistical analysis

In addition to the calculations of descriptive statistics (mean, median, mode), the Kolmogorov-Smirnov test was used to examine the adjustment of the distribution of scores to the normal distribution. The Student *t* test was used to analyze the differences between scores of two independent samples. The association relationships among variables were performed using the Spearman correlation coefficient (r_s_), as it does not require the data to come from a normal distribution, and the intensity of the association was classified as very low if the correlation was < 0.2, low between 0.2 and 0.39, moderate between 0.4 and 0.69, high between 0.7 and 0.89, and very high between 0.9 and 1 [37]. Statistical analyzes were considered significant at *p* < 0.05 (two-tailed tests).

Statistical treatment was performed using the IBM *SPSS Statistics 21.0* program.

## Results

Of the 324 study participants 42.3% were men, the mean age was 27.7 years (SD = 9.84) and the majority were unmarried (74.1%) and mean schooling was 13.0 years (Table 1).

**Table 1:** Sample Characterization

	M + W N = 324	Men *N* = 137	Women *N* = 187	Statistical tests
Age. Mean (SD)	27.7 (9.84)	27.8 (10.01)	27.5 (9.74)	*t* = 0.260 (df = 322) NS
Marital status. N° (%)				
Unmarried	240 (74.07)	106 (77.37)	134 (71.66)	
Married	71 (21.91)	26 (18.98)	45 (24.06)	
Divorced/Widow	13 (4.01)	5 (3.65)	8 (4.28)	χ^2^ = 2.016 (df = 3) NS
Schooling (years)				
Mean (SD)	13.03 (3.18)	12.9 (3.14)	13.1 (3.22)	*t* = 0.629 (df = 322) NS

*t* t Student, χ2= Chi square, *NS* Non-significant

The Global Seasonality Score (GSS), subscale of the Seasonal Pattern Assessment Questionnaire (SPAQ), shows an average score of 9.06 (SD = 4.275), in a range between the minimum and maximum score of 0–21; the other two measures of central tendency, median = 9 and mode = 7, are also to the left of the central point of the scale, reflecting a greater accumulation of results in the lower score. The Kolmogorov-Smirnov adjustment test (F (324) = 0.076 *p* < 0.001) shows that the GSS distribution does not follow a Gaussian (normal) distribution.

The mean score on the Screening Scale for Mental Health (ER80) was 2.35 (SD = 2.736), with a median = 1.50 and mode = 0. Also on this scale the higher frequency of results occurs in the lower score levels, reflecting a deviation from the normal distribution (F (324) = 0.195 *p* < 0.001).

Analyzing the GSS and ER80 values, it shows a positive and significant association between the scores of the two scales, both for the total GSS score and for each of its six items, although only the total score and the item “energy level” the correlation coefficient is > 0.3 (Table 2).

**Table 2:** Correlation between GSS and ER80

	ER80
GSS – Total	0.308 ^**^
Sleep	0.117 ^*^
Social activity	0.192 ^**^
Mood	0.288 ^**^
Body weight	0.183 ^**^
Appetite	0.209 ^**^
Energy level	0.329 ^**^

^*^ *p* < 0.05

^**^ *p* < 0.01

Based on the GSS and responses to perceived seasonal changes as a problem, according to the Kasper criteria (Table 3), 12.7% of the respondents fall within in the Seasonal Affective Disorder (SAD) criteria, 29.0% in subsyndromal seasonal affective disorder (s-SAD) and 58.3% did not show significant seasonal affective symptomatology (without SAD).

**Table 3:** Global Seasonality and Severity Perception Score

	Seasonal Affective Disorder^c^		
	Without SAD	Subsyndromal (s-SAD)	SAD
GSS^d^ ≤ 8	146 (45.1%)	–	–
GSS 9 or 10			
problem <2^a^	43 (13.3%)	–	–
problem ≥2^b^	–	10 (3.1%)	–
GSS ≥ 11			
problem <2^a^	–	84 (25.9%)	–
problem ≥2^b^	–	–	41 (12.7%)
Total	189 (58.3%)	94 (29.0%)	41 (12.7%)

^a^ Seasonal changes experienced as a mild problem

^b^ Seasonal changes experienced as a moderate to severe problem

^c^ Kasper criteria, regardless of the seasonal pattern in which it occurs

^d^ Global Seasonality Score

The comparison of the responses to the Seasonal Pattern Assessment Questionnaire (SPAQ) with the presence of psychiatric pathology evaluated by the ER80 cut-off point (Table 4), shows that the highest concentration occurs in 10.5% individuals with potential psychiatric disease (15 cases), whereas subjects without seasonal affective disorder, evaluated by SPAQ, there were only 6 cases of psychiatric morbidity, respectively, covering 4.6 and 1.9% of the total sample, showing a statistical significant difference (χ^2^ = 41.610 (df = 2) *p* < 0.001).

**Table 4:** Seasonal Affective Disorder and psychiatric morbidity

SPAQ	ER80	
	No	Yes
Seasonal Affective Disorder (SAD)	26 (63.4%)	15 (36.6%)
Subsyndromal seasonal affective disorder (s-SAD)	81 (86.2%)	13 (13.8%)
Without Seasonal Affective Disorder (without SAD)	183 (96.8%)	6 (3.2%)
Total	290 (89.5%)	34 (10.5%)

χ^2^ = 41.610 (df = 2) *p* < 0.001

According to the ER80 criteria, 15 subjects showed scores equal to or greater than four on the falsehood scale. Of these, 12 (80.0%) were subjects without seasonal affective disorder and 3 (20.0%) were included in the subgroup of likely subsyndromal seasonal affective disorder, and there were no cases among those with seasonal affective disorder.

## Discussion

The Seasonal Profile Assessment Questionnaire (SPAQ) is probably the most widely used instrument for evaluating seasonal affective disorder, both in epidemiological and clinical studies, and many studies point to it as a reference tool [6, 10, 21, 27]. However, although its reliability and consistency are recognized, its use remains controversial because it can overestimate the prevalence of seasonal depression [23, 29–31].

In this study, according to the criteria defined by Kasper et al. [22], 12.7% of the individuals in the sample showed likely seasonal affective disorder and 29.0% for subsyndromal seasonal affective disorder. These values are above the upper limits indicated in epidemiological studies based on the SPAQ that refer to the general population prevalence rates of SAD between 0.7 and 12.4% [9, 6, 33, 38], and 2.6 to 19.1% for s-SAD [9, 33]. In this study the sample was selected by non-probabilistic methods that may lead to potential biases. Composed of young subjects, these are pointed out in several studies as having a higher prevalence of seasonal affective variations without a clear explanation for this phenomenon [9, 23, 27]. On the other hand, the fact that they are students undergoing university and professional courses, subject, in the first case, to social rhythms marked by periods of work and vacations, and unemployment, in the second case, this can represent factors that are reflected in the variations of mood and energy.

In Portugal, there are no epidemiological studies on the prevalence of SAD, but a international study showed that Portugal is the second country in Europe with the highest prevalence of psychiatric disorders (22.9%) only surpassed by Northern Ireland (23,1%) [39, 40] and maintains as one of the EU countries with the highest prevalence of depression [41]. Recently, two studies carried out in the French regions of proximal latitudes for the screening of seasonal depression in primary care users using SPAQ showed a prevalence of 14.7 and 24.3% [42, 43], reflects the great variability of results achieved with this instrument. As a self-assessment questionnaire, SPAQ does not diagnose seasonal affective disturbance per se, it only leads to an estimate based on the self-assessment of seasonal variation to a set of six symptoms (mood, appetite, weight, sleep, energy, social activity). Faced with more restrictive criteria (e.g. those defined in successive DSM reviews), it is accepted that SPAQ tends to overestimate seasonal affective disorder [6, 15, 31, 44] and both the specificity and its predictive value have been criticized [23, 45, 46].

According to the initial hypothesis under study, the perception of seasonal affective symptoms corresponds to the perception of worse health status and psychiatric morbidity. In fact, in the subjects with likely SAD and s-SAD were registered, respectively, 36.6 and 13.8% of psychiatric morbidity cases and only 3.2% in non-SAD subjects. It is a relationship that highlights the importance of paying attention to complaints of mood and behavior changes related to the seasons. However, for the analysis of these results, some questions ought to be considered:

First, SPAQ – specifically the GSS subscale – only signal the seasonal changes to a limited set of symptoms, quantifying them according to the intensity and if they are an issue. Based on retrospective self-assessments, it is subject to memory biases that affect this type of instrument and may be influenced by factors such as the emotional significance of the episodes under study, socially desirable patterns of responses, personal characteristics of the respondents, etc. [47–49] Secondly, SPAQ does not take into account the full extent of the diagnostic criteria for depression [23, 30, 31] and although SAD presents as a spectrum of severity [21, 42], some authors consider that SAD represents a moderate variant of depression, characterized by an atypical profile of symptoms, with lower cognitive and occupational impairment than non-seasonal depression [50], a common phenomenon in the population, as reflected in the high values ​​of the evaluation with SPAQ.

Thirdly, the SPAQ-GSS does not verify the presence of episodes of depression, but the perception of seasonality in mood changes and behavior in an extended period of time [23, 44]. In turn, the ER80 questionnaire investigates the presence of psychopathological symptoms in the period prior to the survey in order to identify cases of psychiatric morbidity. The two questionnaires cover different time windows and, in the case of a cross-sectional study, it may happen that some subjects at the time of responding do not meet the conditions to be designated as psychiatric cases (ER80), although they may have had typical mood fluctuations of disturbance during the time leading up to the survey.

In turn, the ER80 seeks to identify cases of psychiatric morbidity, regardless of its nature, so it encompasses more situations than depressive states. This fact explains that 3.2% of subjects without SAD scored positively for psychiatric morbidity and among subjects with SAD there may be cases of psychiatric co-morbidity with other disorders than depression.

Given these issues, this study reveals that subjects with complaints of mood and behavior changes related to the seasons, inscribed in the concept of SAD and s-SAD demonstrate a very significant number of cases with clinically significant psychiatric morbidity. Considering that this is a study carried out in a non-clinical population, this situation draws attention to the importance of the knowledge of this entity and to its early diagnosis and treatment.

## Conclusion

In the sample studied, composed of students undergoing higher education and vocational courses, the seasonal affective disorder, evaluated through the SPAQ questionnaire, presented a high prevalence, with a statistically significant association with states of psychiatric morbidity. Subjects with SAD and s-SAD showed 36.6 and 13.8% cases of psychiatric morbidity respectively, whereas in subjects without SAD, this value was only 3.2%.

The finding of psychiatric morbidity in a significant number of subjects with seasonal fluctuations in mood and behavior points to the need for its recognition and importance of clinical follow-up. Three decades after the first descriptions of seasonal affective disorder, it still remains a controversial entity, but the suffering caused by depressive states and the existence of effective therapeutic measures justify the continuation of studies that broaden the understanding of this disorder and contribute to reduce the negative consequences and improve the patients’s quality of life. In this sense, one of the challenges is the study of the use of light therapy as a therapy to prevent depressive episodes in people with susceptibility to seasonal variations. This non-pharmacological treatment has a therapeutic potential that needs to be further investigated, namely in the direction now pointed out, with prospective and controlled studies.

## Supplementary Information

**Additional file 1.**

## Data Availability

All the data supporting our findings have been presented in the manuscript; the datasets used and/or analyzed during the current study are available from the corresponding author on request.

## Abbreviations

SAD: Seasonal affective disorder
s-SAD: Subsyndromal seasonal affective disorder
SPAQ: Seasonal Pattern Assessment Questionnaire
GSS: Global Seasonality Score
ER80: Screening Scale for Mental Health
