# Supplementary material for: Seasonal sensitivity and psychiatric morbidity: study about seasonal affective disorder
Source: BMC Psychiatry. 2021 Jun 29;21:317. doi: 10.1186/s12888-021-03313-z (PMC8243845; doi:10.1186/s12888-021-03313-z)
Supplement: Supplementary file 1 — Additional file 1. [file 12888_2021_3313_MOESM1_ESM.docx]

Supplementary information

Screening Scale for Mental Health (ER80). Translation of the items:

Have you been experiencing headaches? (often/few/never)

Have you been feeling ill? (often/few/never)

When you feel sick, do you always seek for medical help? (yes/no)

Have you been feeling nervous or irritable? (often/few/never)

Have you been feeling satisfied or sad? (satisfied/normal/sad)

How has your memory been? (good/regular/weak)

Have you been feeling your head heavy? (yes/rarely/no)

Do you have the feeling that everything goes wrong? (yes/no)

Do you find it hard to decide about little things in your everyday life? (yes/rarely/no)

Have you been able to sleep without any difficulty? (yes/almost always/no)

Have you been feeling worried about useless things? (yes/no)

Have you had any trouble paying attention to a conversation or TV show? (yes/no)

Do you think people have been treating you differently? (yes/no)

Is there anyone you dislike? (yes/no)

Do you feel better when you are alone? (yes/no)

Do strange or unexplained thing have been happening to you? (yes/no)
